# Long Non-coding RNA LINC00115 Contributes to the Progression of Colorectal Cancer by Targeting miR-489-3p via the PI3K/AKT/mTOR Pathway

**DOI:** 10.3389/fgene.2020.567630

**Published:** 2020-09-16

**Authors:** Weiyu Feng, Baodong Li, Jinbang Wang, Huiliang Zhang, Yonggang Liu, Dongli Xu, Ke Cheng, Jing Zhuang

**Affiliations:** Department of General Surgery, The Affiliated Cancer Hospital of Zhengzhou University, Henan Cancer Hospital, Zhengzhou, China

**Keywords:** long non-coding RNA, LINC00115, colorectal cancer, metastasis, miR-489-3p

## Abstract

Long non-coding RNAs (lncRNAs) are tumor-related regulators and have been found to be involved in the underlying molecular mechanisms of colorectal cancer (CRC). However, the role of lncRNA LINC00115 during CRC progression is not entirely elucidated. In this study, we discovered that LINC00115 was significantly overexpressed in CRC, and its overexpression predicted poor patient outcomes. Downregulation of LINC00115 markedly inhibited CRC cell proliferation, increased cell apoptosis, and suppressed cell migration and invasion. Moreover, downregulation of LINC00115 led to the inactivation of PI3K/AKT/mTOR signaling. Bioinformatics analysis identified miR-489-3p as a candidate target of LINC00115. Furthermore, we revealed an inverse correlation between LINC00115 and miR-489-3p in CRC tissues. Importantly, by luciferase reporter assay, we found that miR-489-3p might directly target LINC00115, and downregulation of miR-489-3p could rescue the biological effects induced by the absence of LINC0015. In conclusion, our findings demonstrated that LINC00115 serves as an oncogene in CRC metastasis. Deeper understanding of the LINC00115/miR-489-3p axis might provide potential therapeutic targets against CRC metastasis.

## Introduction

As an aggressive and metastatic disease, colorectal cancer (CRC) is the fifth most common cancer globally and the third most common cause of cancer-related mortality (Siegel et al., 2019). In a recent report by Chen et al., CRC amounts to the second leading cause of cancer-related mortality, and its incidence is still on the rise in China (Chen et al., 2018). Although advances in surgery and chemotherapy have been achieved during the past decades, survival and prognosis are still poor because most patients with advanced CRC present emergent chemoresistance, cancer recurrence, or distant metastasis (Siegel et al., 2017; Dekker et al., 2019). Furthermore, the underlying molecular mechanisms of CRC carcinogenesis and progression have not yet been fully understood. In recent years, it has been reported that CRC develops through the accumulation of genetic mutants and epigenetic modifications (Coppede et al., 2014; Okugawa et al., 2015; Hong, 2018). Thus, it is necessary to develop a better understanding of the development and progression of CRC to identify novel improved therapeutic strategies for CRC patients.

Non-coding RNAs (ncRNAs), including microRNAs (miRNAs), long ncRNAs (lncRNAs), and circular RNAs (circRNAs), have been recently reported to be involved in the regulation of protein-coding genes in both physiological and pathological conditions (Kapranov et al., 2007; Esteller, 2011; Cech and Steitz, 2014). In the CRC carcinogenesis and progression, some miRNAs were reported to play a crucial role in cell function modulation, including cell growth, invasion, autophagy, and apoptosis (Chandra Gupta and Nandan Tripathi, 2017; Dai et al., 2019). For example, miR-708 is reported to be one of the lowest expressed miRNAs in CRC tissues and could suppress cell proliferation, induce apoptosis, and reduce metastasis by directly targeting the Zinnia endonuclease 1. Just like the extensive participation of miRNAs in cancer, lncRNAs, which are transcripts usually longer than 200 nucleotides with limited protein-coding capability, have attracted much more attention due to their unique role during cancer development and progression. Several lncRNAs, such as metastasis-associated lung adenocarcinoma transcript 1, HOX transcript antisense RNA, TTN antisense RNA 1 (TTN-AS1), small nucleolar RNA host gene 6, and long intergenic non-protein coding RNA 1503 (LINC001503) have been demonstrated recently to be involved in the competitive endogenous RNA (ceRNA) network and regulate gene expression in several cancers, including CRC (Lu et al., 2018; Duan et al., 2019; Sun et al., 2019; Wang X. et al., 2019; Wang Y. et al., 2019; Yu et al., 2019). For instance, lncRNA growth arrest-specific 5 upregulated the expression of phosphatase and tensin homolog by functioning as a ceRNA of miR-222-3p, thus inhibiting CRC cell migration and invasion and promoting cell autophagy (Liu L. et al., 2019). It is certain that the interplay between lncRNAs and miRNAs exerts potential roles in CRC carcinogenesis and progression and is critical for the regulatory network of CRC.

In our study, we first detected the expression patterns of LINC00115 in CRC and found LINC00115 to be significantly upregulated. We also found that LINC00115 knockdown significantly inhibited CRC cell proliferation, induced cell apoptosis, and suppressed the metastatic capacity. Bioinformatics analysis revealed a potential interaction between LINC00115 and miR-489-3p. Therefore, we explored the expression profiles of miR-489-3p in CRC tissues and verified its relationship with LINC00115, revealing a promising LINC00115/miR-489-3p axis that contributes to the progression of CRC.

## Materials and Methods

### Tissue Samples

CRC and normal tissue samples were selected from 100 CRC patients treated with radical surgery in the Henan Oncology Hospital between 2012 and 2014. All patients signed the informed consent voluntarily, and their tissue samples had been examined by at least three experienced pathologists. The study was approved by the Medical Ethics Committee of the Affiliated Cancer Hospital of Zhengzhou University, Henan Cancer Hospital.

### Cell Cultures

Five CRC cancer cell lines (LoVo, HT-29, Caco-2, SW620, and SW480) and normal epithelial cell lines (FHC) were obtained from ATCC (Shanghai, China). In addition, Dulbecco’s modified Eagle’s medium (DMEM) (Gibco, Grand Island, NY, United States) supplemented with 10% fetal bovine serum (Gibco, Australia origin) was used for cell incubation. Moreover, these cells were cultured in the incubator at 37°C with 5% CO_2_.

### Cell Transfection

The LINC00115-specific small-interfering RNAs (siRNAs) were applied to transfect the CRC cell lines (including HT-29 and LoVo) with Lipofectamine 3000 transfection reagent (Thermo Fisher Scientific, United States). In addition, miR-489-3p inhibitors or relative negative control sequence (miR-487-3p NC) were further transfected. The LINC00115-1 downregulated CRC cells were further transfected with Lipofectamine 3000 transfection reagent. The complete siRNA sequences are listed in Table 1.

**TABLE 1:** Sequences of oligomers and primers used in the present research.

**Name**	**Sequence (5′–3′)**
si-LINC00115-1	AAG GAA UUU GGG AAU UAG GCU
	CCU AAU UCC CAA AUU CCU UAG
si-LINC00115-2	UUG GAA AUC AGA AAA CCA CUA
	GUG GUU UUC UGA UUU CCA AAU
LINC00115 forward	TGG CTT GTC TTC CAT CGT CC
LINC00115 reverse	GCA CGA GGG TTG TTA CAG GA
miR-489-3p forward	AGG GGG TGA CAT CAC ATA TAC
miR-489-3p reverse	GAG AGG AGA GGA AGA GGG AA
GAPDH forward	CCT TCC GTG TCC CCA CT
GAPDH reverse	GCC TGC TTC ACC ACC TTC

### RT-qPCR Assay

According to the manufacturer’s protocol, the total RNA from the tissue samples or CRC cell lines was extracted with Trizol reagent (Takara, Dalian, China). Then, the PrimeScript RT Master Mix (Takara) was used to reverse transcribe 500 ng RNA into complementary DNA (cDNA), followed by the cDNA amplification with SYBR-Green PCR kit (Roche, Basel, Switzerland). Reactions were incubated in a 96-well optical plate at 95°C for 10 min, followed by 40 cycles at 95°C for 15 s and 60°C for 60 s. The expression levels of the target genes were analyzed with the 2^–ΔΔCt^ method. The complete sequences of the primes are shown in Table 1.

### Cell Counting Kit 8 Assay

First, the treated cells (1000/well) were collected and planted in 96-well plates. After incubation for 24, 48, 72, and 96 h, diluted Cell Counting Kit 8 (CCK-8) solution was added into each well for reaction, respectively. Subsequently, the optical density value was collected by a microplate reader (Molecular Devices, Sunnyvale, CA, United States) at 450 nm.

### Transwell Assay

The Transwell chambers (0.8 μm; Corning, NY, United States) with or without Matrigel coating (Corning) were used to evaluate cell migration and invasion ability. First, the treated cells (3 × 10^5^/well) were collected and placed in the top chamber, while 500 μl of DMEM medium with 20% fetal bovine serum was added in the low chamber. Subsequently, after removing the non-invading and non-migrating cells with cotton-tipped swabs, the other cells were fixed and stained with 0.1% crystal violet. Finally, the invaded and migrated cells were examined with a light microscope.

### Flow Cytometric Analysis

For cell apoptosis rate detection, cells were treated with a cell apoptosis detection kit (Keygen, Nanjing, China) and were evaluated by a FACSCanto II flow cytometer (San Jose, CA, United States) according to the manufacturer’s instructions.

### Dual-Luciferase Reporter Assays

Cells were transfected with pmirGLO-LINC00115-MUT or pmirGLO-LINC00115-WT plasmid and then cotransfected with miR-489-3p mimics or miR-489-3p NC. Then, the relative luciferase activity was detected and evaluated by the dual-luciferase reporter assay system (Promega, Madison, WI, United States) according to the manufacturer’s instructions.

### Western Blotting

Radioimmunoprecipitation assay (RIPA) lysis buffer (Beyotime, Shanghai, China) was applied to extract the total proteins. Subsequently, the total protein concentration was detected and evaluated via the BCA Protein Assay Kit (Beyotime). Then, equal quantities of proteins were separated on 10% sodium dodecyl sulfate–polyacrylamide gel electrophoresis (SDS-PAGE) gel and transferred into polyvinylidene fluoride membranes. Subsequently, the membranes were incubated with 10% bovine serum albumin and then with primary antibodies (PI3K, p-PI3K, AKT, p-AKT, mTOR, p-mTOR, and GADPH) and then incubated with secondary antibodies. Finally, GeneSnap using SynGene systems was performed to evaluate the protein bands.

### Bioinformatics Analysis

The binding site of LINC00115 and miR-489-3p was predicted using the starBase platform^1^.

### Statistical Analysis

All data were shown as mean ± SD. Then, GraphPad Prism v7.0 software and IBM SPSS 20.0 software were applied for data evaluation in this study. All experiments were performed at least three times. Furthermore, data differences between the two groups were evaluated by chi-square test or Student’s *t-*test. For *p* < 0.05, the differences were considered statistically significant.

## Results

### LINC00115 Is Upregulated in CRC

**FIGURE 1:**
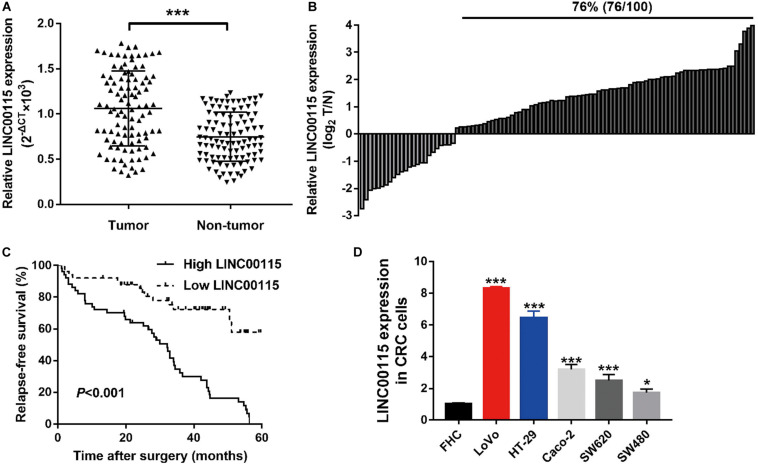
LINC00115 is upregulated in colorectal cancer (CRC). **(A)** LINC00115 was significantly upregulated in CRC tissues samples relative to normal tissues evaluated by reverse transcription quantitative PCR (RT-qPCR) assay. **(B)** LINC00115 is overexpressed in 76% (76/100) cases of CRC patients. **(C)** The relationship between the expression of LINC00115 and relapse-free survival time. **(D)** LINC00115 was upregulated in CRC cell lines (LoVo, HT-29, Caco-2, SW620, and SW480) relative to the normal cells (FHC). *p < 0.05, ***p < 0.001.

The expression pattern of LINC00115 in CRC tissues was detected using reverse transcription quantitative PCR (RT-qPCR assay), and LINC00115 was found to be dramatically upregulated in CRC tissue samples (Figure 1A, *p* < 0.001). Meanwhile, in this study cohort, cases with LINC00115 overexpression accounted for 76% (76/100) of the CRC patients (Figure 1B). Subsequently, the tissue samples cohort was divided into low- and high-expression groups, based on the median of relative LINC00115 expression. Further statistical analysis revealed that expression of LINC00115 was highly associated with T stage (*p* = 0.000), N stage (*p* = 0.000), and tumor–node–metastasis (TNM) stage (*p* = 0.000) (Table 2). In addition, we performed multivariate analysis with Cox regression model to evaluate the prognostic correlation between the LINC00115 expression and the clinicopathological characteristics of CRC patients. The results showed that TNM stage (*p* = 0.000) and LINC00115 expression (*p* = 0.000) were highly related with relapse-free survival time of CRC patients (Figure 1C), while multivariate analysis showed that TNM stage (95% CI: 2.308–22.954, *p* = 0.001) and LINC00115 expression (95% CI: 1.203–5.108, *p* = 0.014) might be the independent risk factors for CRC patients in relapse-free survival (Table 3). Taken together, our results indicated that LINC00115 was upregulated in CRC tissues, and its expression was closely associated with the pathogenesis and outcomes of CRC patients.

**TABLE 2:** Correlation between LINC00115 expression and clinicopathological characteristics of patients with colorectal cancer (*n* = 100).

**Variables**	**LINC00115 expression**		***p*^#^**
	**High**	**Low**	
**Gender**			
Male	32	25	0.157
Female	18	25	
**Age (years)**			
<60	33	32	0.834
≥ 60	17	18	
**Histological grade**			
Well	19	20	0.296
Moderate	18	23	
Poor	13	7	
**T stage**			
T1 + T2	9	36	0.000
T3 + T4	41	14	
**N stage**			
N0	20	42	0.000
N1–N2	30	8	
**TNM stage**			
I + II	17	40	0.000
III + IV	33	10	
**CEA levels**			
Normal	32	26	0.224
Elevated	18	24	
**CA199 levels**			
Normal	38	30	0.086
Elevated	12	20	

*^#^p < 0.05 indicates statistical significance.*

**TABLE 3:** Univariate and multivariate Cox regression analysis of LINC00115 for predicting the relapse-free survival of patients with colorectal cancer (*n* = 100).

**Variables**	**HR**	**95% CI**	***p*^#^**
**Univariate analysis**			
Gender (male vs. female)	1.240	0.726–2.116	0.431
Age (years) (≥60 vs. <60)	0.866	0.504–1.487	0.602
Histological grade (well, moderate, poor)	–	–	0.225
Histological grade (poor vs. well)	1.661	0.933–2.957	0.085
Histological grade (moderate vs. well)	1.265	0.615–2.603	0.523
T stage (T3 + T4 vs. T1 + T2)	2.524	1.458–4.372	0.001
N stage (N1–N2 vs. N0)	5.685	3.264–9.902	0.000
TNM stage (III + IV vs. I + II)	7.302	4.073–13.091	0.000
CEA levels (elevated vs. normal)	0.829	0.488–1.407	0.487
CA199 levels (elevated vs. normal)	0.713	0.395–1.285	0.261
LINC00115 expression (high vs. low)	3.927	2.152–7.167	0.000
**Multivariate analysis**			
T stage (T3 + T4 vs. T1 + T2)	0.680	0.305–1.518	0.347
N stage (N1–N2 vs. N0)	0.893	0.335–2.376	0.820
TNM stage (III + IV vs. I + II)	7.278	2.308–22.954	0.001
LINC00115 expression (high vs. low)	2.479	1.203–5.108	0.014

*^#^p < 0.05 indicates statistically significant.*

### Downregulation of LINC00115 Inhibits Cell Growth in CRC Cells

**FIGURE 2:**
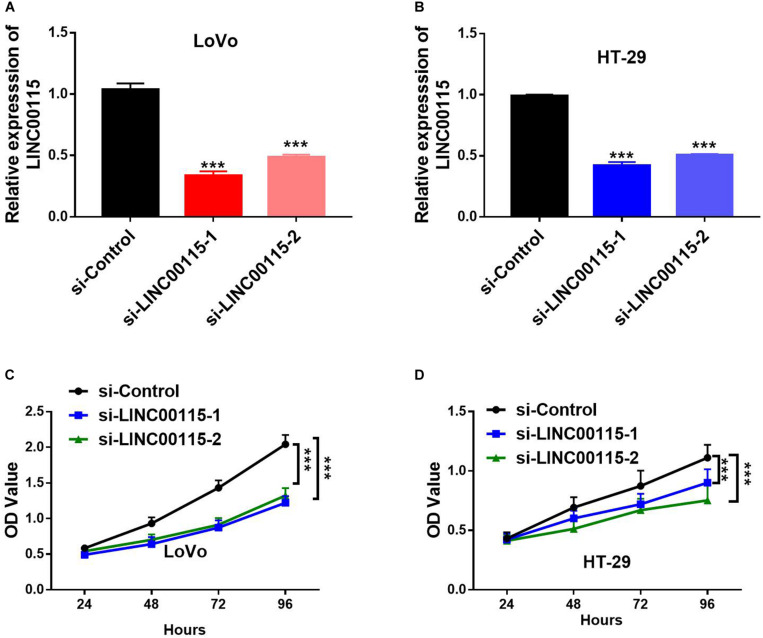
Knockdown of LINC00115 suppresses cell growth on colorectal cancer (CRC) cells. **(A,B)** Downregulation efficiency of LINC00115 by a small interfering RNA (siRNA)-transfected method in LoVo cells and HT-29 cells, respectively. **(C,D)** Cell growth ability of LINC00115-downregulated LoVo cells and HT-29 cells detected by CCK-8 assay. ***p < 0.001.

As shown in RT-qPCR results, LINC00115 is also significantly upregulated in CRC cell lines (LoVo, HT-29, Caco-2, SW620, and SW480 cells) when compared with the normal cell (FHC) (Figure 1D). LoVo and HT-29 cells expressed higher LINC00115 levels. Then, we constructed LINC00115 knockdown LoVo and HT-29 cells via transfection of two independent siRNA sequences. The results revealed that compared to a negative control group (si-Control), the selected siRNAs could significantly downregulate LINC00115 expression in LoVo and HT-29 cells (referred to si-LINC00115-1 group and si-LINC001156-2 group), while si-LINC00115-1 group showed higher inhibition efficiency (Figures 2A,B). CCK-8 assay revealed that the silencing of LINC00115 could significantly suppress the proliferative ability of LoVo and HT-29 cells (Figures 2C,D). Consequently, our data revealed that LINC00115 was closely implicated in the CRC cell growth.

### Downregulation of LINC00115 Induces Cell Apoptosis in CRC Cells

**FIGURE 3:**
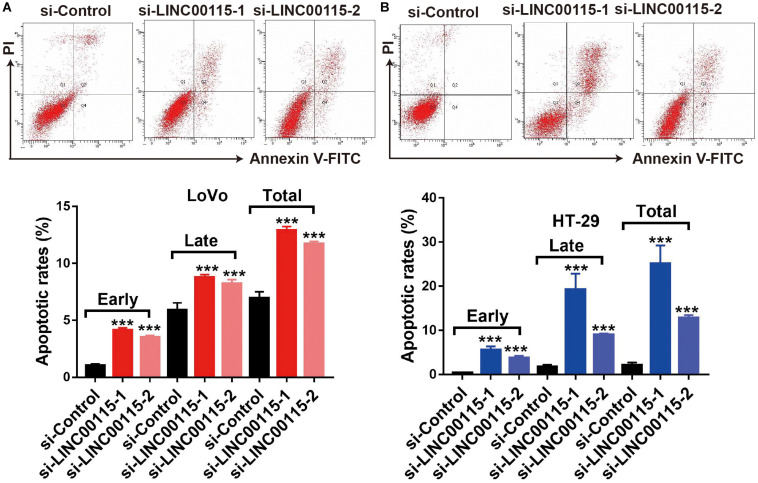
Downregulation of LINC00115 induces more apoptosis in colorectal cancer (CRC) cells. **(A)** Cell apoptotic images of LoVo cells and transfected with LINC00115-specific siRNAs (up) and the relative statistical analysis (down). **(B)** Cell apoptotic images of HT-29 cells transfected with LINC00115-specific siRNAs (up) and the relative statistical analysis (down). ***p < 0.001.

Since CRC cell apoptotic status is highly associated with proliferative ability, we further detected the apoptosis rates of CRC cells using flow cytometry. Compared with cells in the control group, LoVo cells in the si-LINC00115-1 and the si-LINC00115-2 groups exhibited an increased apoptotic rate, including early, late, and total proportions (Figure 3A). Similar results were observed in HT-29 cells, where downregulation of LINC00115 by siRNAs induced increased early, late, and total cell apoptosis (Figure 3B). Therefore, our data suggested that LINC00115 could induce CRC cell apoptosis *in vitro*.

### Downregulation of LINC00115 Inhibits Metastasis in CRC Cells

**FIGURE 4:**
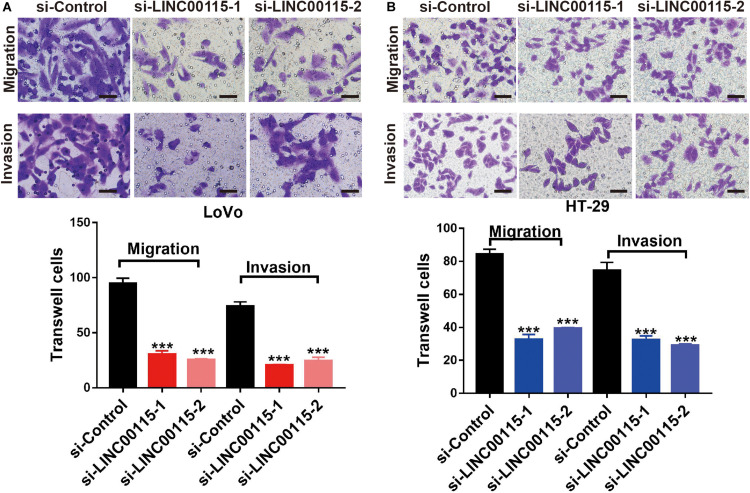
Downregulation of LINC00115 suppresses migration and invasion abilities of colorectal cancer (CRC) cells. **(A)** Knockdown of LINC00115 dramatically reduced the number of invaded and migrated LoVo cells detected by Transwell assay. **(B)** Knockdown of LINC00115 dramatically reduced the number of invaded and migrated HT-29 cells detected by Transwell assay. ***p < 0.001.

Considering that patients with LINC00115 dysregulation always presented with lymph node metastasis, we speculated that LINC00115 might play a pivotal role in CRC cell metastasis. As shown in the Transwell assay, LINC00115 silencing significantly inhibited cell migration and invasion abilities of LoVo cells (Figure 4A). Consistently, HT-29 cells in si-LINC00115-1 and si-LINC00115-2 groups showed decreased migration or invasion counts compared with cells in the control group (Figure 4B). Hence, our data indicated that LINC0015 downregulation inhibits metastasis in CRC cells.

### Downregulation of LINC00115 Regulates the PI3K/AKT/mTOR Pathway in CRC Cells

**FIGURE 5:**
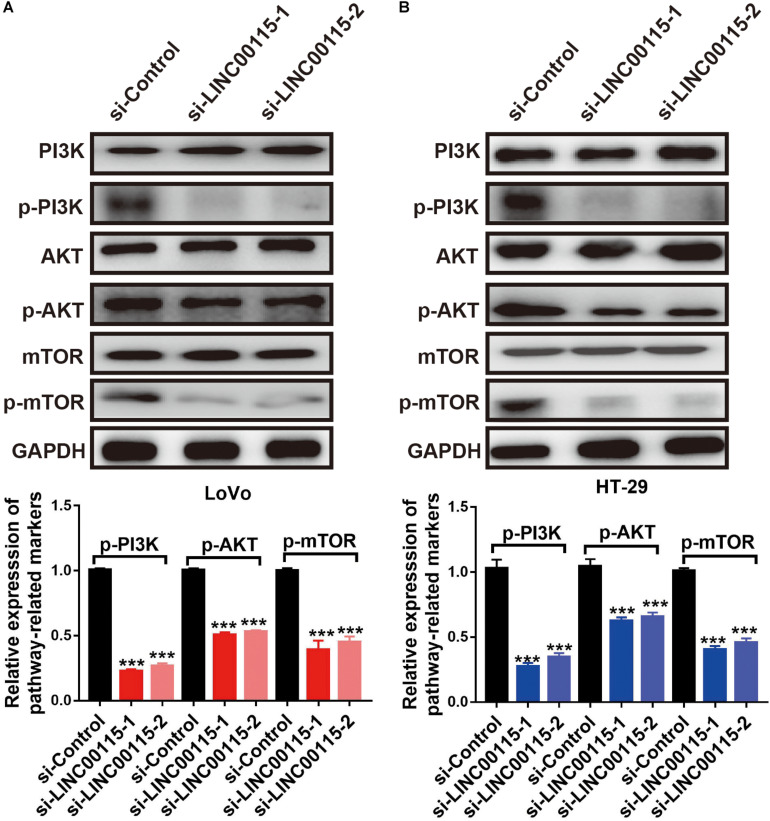
Downregulation of LINC00115 regulates PI3K/AKT/mTOR pathway in colorectal cancer (CRC) cells. **(A)** After transfected with LINC00115-specific small interfering RNAs (siRNAs), the expression levels of PI3K, AKT, mTOR, p-PI3K, p-AKT, and p-mTOR in LoVo cells (up) and the relative gray value (down). **(B)** After transfected with LINC00115-specific siRNAs, the expression levels of PI3K, AKT, mTOR, p-PI3K, p-AKT, and p-mTOR in HT-29 cells (up) and the relative gray value (down). ***p < 0.001.

To clarify how LINC00115 regulates the proliferation and metastasis of CRC cells, we performed Western blotting and found that silencing LINC00115 in LoVo cells could significantly reduce the expression levels of p-mTOR, p-PI3K, and p-AKT proteins (Figure 5A). In line with this observation, the reduction in p-mTOR, p-PI3K, and p-AKT proteins was also observed in HT-29 cells with si-LINC00115-1 or si-LINC00115-2 transfection (Figure 5B). Therefore, we concluded that LINC00115 downregulation might suppress metastatic and proliferative abilities through the PI3K/AKT/mTOR pathway in CRC.

### MiR-489-3p Is a Direct Downstream Target of LINC00115 in CRC

**FIGURE 6:**
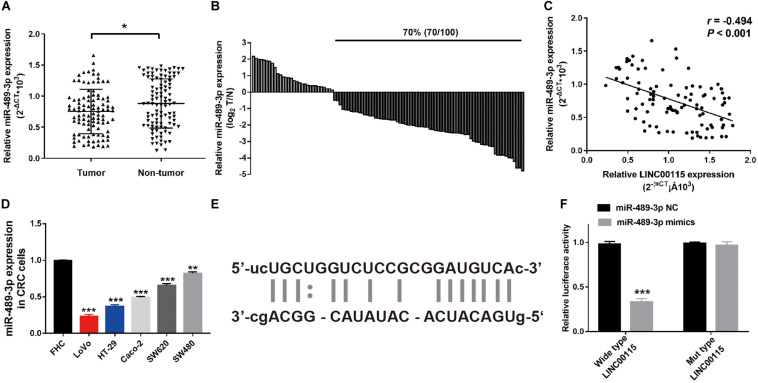
MiR-489-3p might be the downstream of LINC00115. **(A)** MiR-489-3p was significantly upregulated in colorectal cancer (CRC) tissues relative to adjacent normal tissues detected by reverse transcription quantitative PCR (RT-qPCR). **(B)** MiR-489-3p is downregulated in 70% (70/100) of CRC patients. **(C)** Correlation between LINC00115 and miR-489-3p in 100 cases of CRC tissue samples. **(D)** The expression of miR-489-3p in CRC cell lines and the normal cells. **(E)** The predicted 3′ UTR binding regions of LINC00115 on miR-489-3p. **(F)** Relative luciferase activity in 293T cells after cotransfection with pmirGLO-LINC00115-WT or pmirGLO-LINC00115-MUT, along with miR-489-3p specific mimics or NC. *p < 0.05, **p < 0.01, ***p < 0.001.

Using a publicly available bioinformatic algorithm (starBase v2.0), we identified that miR-489-3p might be a downstream target of LINC00115. To further address the relationship between miR-489-3p and LINC00115 in CRC progression, we first detected the expression pattern of miR-489-3p in CRC samples. RT-qPCR results showed that, compared with normal tissue, miR-489-3p was significantly downregulated in CRC tumor samples (Figure 6A, *p* < 0.05), and the downregulation of miR-489-3p accounted for 70% (70/100) of the CRC tissue samples (Figure 6B). Interestingly, we also found that the expression levels of miR-489-3p were negatively correlated with those of LINC00115 (Figure 6C). Meanwhile, RT-qPCR results also showed that, compared with normal cells (FHC), miR-489-3p is significantly downregulated in CRC cell lines (Figure 6D); LoVo and HT-29 cells also showed a lower expression (Figure 6D). To clarify the underlining association, we subsequently predicted their potential binding site, which is shown in Figure 6E. Further luciferase reporter assay indicated that, when treated with miR-489-3p mimics, wild-type cells showed lower luciferase activity (Figure 6F). Therefore, these results consistently revealed that miR-489-3p might be a direct target of LINC00115 in CRC.

### MiR-489-3p Inhibition Rescues the CRC Cells Proliferation Ability Induced by the LINC00115 Depletion

**FIGURE 7:**
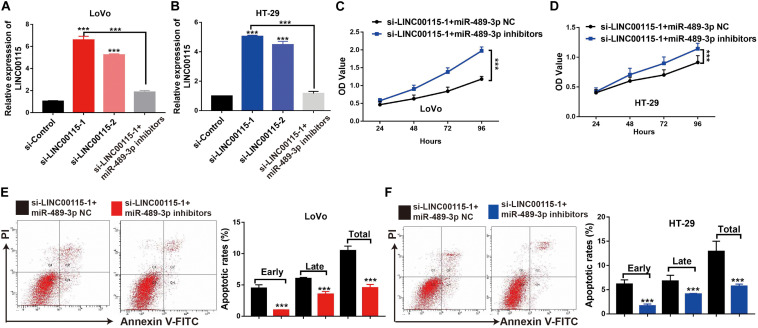
Inhibition of miR-489-3p rescue anticell proliferation abilities colorectal cancer (CRC) cells induced by depletion of LINC00115 in CRC cells. **(A,B)** The expression of miR-489-3p in LINC00115-downregulated LoVo and HT-29 cells after transfection with miR-489-3p negative control (NC) or miR-489-3p inhibitors. **(C,D)** Cell proliferation ability of LINC00115-downregulated LoVo and HT-29 cells, with or without depletion of miR-489-3p, detected by CCK-8 assay. **(E,F)** Cell apoptotic rates of LINC00115-downregulated LoVo and HT-29 cells, with or without depletion of miR-489-3p, detected by flow cytometry. ***p < 0.001.

First, we discovered that, compared with the si-control group, the si-LINC00115-1 and si-LINC00115-2 groups showed higher expressions of miR-489-3p, and the si-LINC00115-1 group showed the highest expression of miR-489-3p (Figures 7A,B). To further confirm the potential role of miR-489-3p in regulating CRC progression, we downregulated the expression of miR-489-3p in si-LINC00115-1 LoVo and HT-29 cells by transfecting miR-489-3p specific inhibitors (Figures 7A,B). CCK-8 assay showed that miR-489-3p inhibition could increase cell proliferation ability in LINC00115-knockdown LoVo and HT-29 cells (Figures 7C,D). Furthermore, flow cytometric analysis showed that miR-489-3p downregulation could significantly reduce cell apoptosis rate when compared with miR-489-3p NC group (Figures 7E,F). These results consistently indicated that downregulation of LINC00115 might suppress CRC cell growth by targeting miR-489-3p.

### MiR-489-3p Inhibition Rescues the CRC Cells Metastatic Ability and the PI3K/AKT/mTOR Signaling Pathway Is Induced by the LINC00115 Depletion

**FIGURE 8:**
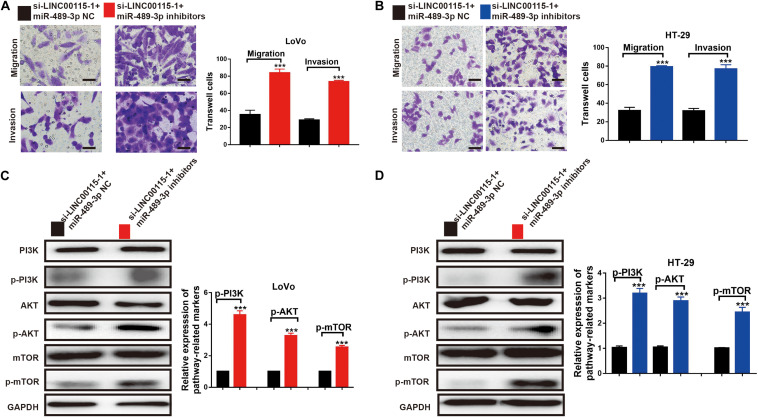
Inhibition of miR-489-3p reverse the anticell metastasis abilities and PI3K/AKT/mTOR pathway induced by depletion of LINC00115 in colorectal cancer (CRC) cells. **(A,B)** Knockdown of LINC00115 dramatically reduced the number of invaded and migrated LoVo and HT-29 cells detected by Transwell assay. **(C,D)** The expression levels of PI3K, AKT, mTOR, p-PI3K, p-AKT, and p-mTOR on LoVo and HT-29 cells (up) and the relative gray value (down). ***p < 0.001.

The results of the Transwell assays showed that, both in LoVo (Figure 8A) and HT-29 cells (Figure 8B), the antimetastatic ability induced by the LINC00115 depletion could be reversed by miR-489-3p inhibition. Furthermore, after the miR-489-3p depletion, the expression levels of p-mTOR, p-AKT, and p-PI3K proteins in si-LINC00115-1 LoVo and HT-29 cells were significantly increased compared with those in the miR-489-3p NC group (Figures 8C,D). In summary, these results consistently indicated that LINC00115 downregulation could suppress the CRC cells’ metastatic ability and regulate the PI3K/AKT/mTOR signaling pathway.

## Discussion

An increasing number of studies have highlighted the critical roles of lncRNAs in CRC carcinogenesis and cancer cells metastasis. They could serve as a multifaceted regulator in a broad range of cancer gene modulation in transcriptional, posttranscriptional, and epigenetic levels. Here, we reported for the first time the lncRNA LINC00115 as a novel cancer-promoted regulator in CRC. LINC00115 was originally reported in lung cancer as a potential prognostic biomarker (Jiang et al., 2018). In another study, LINC00115 was identified to be a critical regulator of glioma stem-like cell tumorigenicity (Tang et al., 2019). In our study, qRT-PCR assay was conducted to estimate the LINC00115 expression levels in 100 paired CRC tissues and adjacent normal tissues. The results showed a significantly increased LINC00115 expression in CRC tissues compared to adjacent normal tissues. We also observed that upregulation of LINC00115 was strongly related to the advanced TNM stage, larger tumor size, and lymphatic metastasis of CRC patients, thus suggesting that LINC00115 could be a prognostic factor for CRC.

There is increasing evidence that a high lncRNA expression is significantly correlated with unfavorable CRC prognosis. The elevated expressions of lncRNA TTN-AS1, XIST, TUG1, and SNHG12 indicate poor prognoses for CRC patients (Liu et al., 2018; Liu Y. et al., 2019; Wang M. et al., 2019; Wang Y. et al., 2019). Our multivariate survival study proved that LINC00115 is an independent prognostic factor for relapse and poor survival in CRC patients. Importantly, functional assays further identified that knockdown of LINC00115 could have inhibited cell proliferation, migration, and invasion and facilitated cell apoptosis *in vitro*. These findings indicate that LINC00115 was associated with the CRC carcinogenesis and progression, but the exact regulatory mechanism still needs to be clarified.

An increasing amount of multiscale omics data showed that lncRNAs exert their biological effects by working together with different molecules, such as miRNAs, mRNAs, and proteins. For instance, lncRNAs might interact with miRNAs by binding onto their 3′ untranslated region and thus acting as miRNA sponges. Currently, the functional pattern of LINC00115 in CRC cells remains to be established. Hence, we searched several databases and found that miR-489-3p was a target gene for LINC00115. Previous research has shown that miR-489-3p was sponged by LINC01446 and targeted TPT1 to regulate glioblastoma progression (Zhang et al., 2018). In osteosarcoma, miR-489-3p significantly suppressed cell invasion and metastasis both *in vitro* and *in vivo* (Liu et al., 2017). In the present study, we are the first to propose that miR-489-3p might be a target of LINC00115. Through the luciferase reporter gene assay, we confirmed that LINC00115 could directly bind to miR-489-3p. We also found a negative correlation between LINC00115 and miR-489-3p expressions in CRC tissues. Therefore, we predicted that LINC00115 acts as a ceRNA to sequester miR-489-3p, but further experiments are needed.

Numerous studies collectively indicated the importance of intricate crosstalk between lncRNAs and PI3K/AKT/mTOR signaling (Zhu et al., 2016; Huang et al., 2019). mTOR frequently acts as an oncogenic signaling cascade in human malignancies (Hua et al., 2019). It is a serine/threonine protein kinase belonging to the PI3K-related kinase family and forms the catalytic subunit of two kinds of protein complexes, including mTOR complex 1 (mTORC1) and 2 (mTORC2) (Tsang et al., 2007). While mTORC1 controls gene transcription and protein translation in growth-related processes, mTORC2 promotes cell proliferation and survival. Accumulating studies have revealed that PI3K/AKT/mTOR acted as key drivers of cellular growth, adhesion, migration, and survival in human carcinogenesis, including CRC, in which the activation of PI3K/AKT/mTOR signaling supports cancer cell growth, metastasis, and drug resistance (Meng and Zheng, 2015; Bahrami et al., 2018). PI3K/AKT/mTOR signaling also serves as an integration mediator in the crosstalk of oncogenic signaling pathways (Bahrami et al., 2018). PI3K and AKT phosphorylation is the key step for their activation. Once phosphorylated, PI3K activates and phosphorylates AKT and mTOR downstream to cause a cascade reaction. In recent studies, miR-489-3p was shown to interact with the PI3K/AKT/mTOR signaling in various cancers, such as glioma, breast cancer, and melanoma (Chen et al., 2016, 2017; Li et al., 2017). Interestingly, it was found that the phosphorylated levels of PI3K (p-PI3K), AKT (p-AKT), and mTOR (p-mTOR) were decreased after LINC00115 depletion, while their total protein levels remained unchanged, suggesting that LINC00115 positively regulates the PI3K/AKT/mTOR pathway. The involvement of LINC00115 in mediating CRC cell proliferation and migration via modulating the PI3K/AKT/mTOR pathway justifies the idea that anti-LINC00115 compounds or agents consequently targeting the PI3K/AKT/mTOR pathway might serve as novel therapeutic strategies for the CRC treatment.

## Conclusion

In conclusion, our study has delineated the unique role of LINC00115 in CRC and specifically described the underlying molecular mechanisms. We confirmed that LINC00115 is upregulated in CRC and functions as an independent predictor of progression-free survival, leading to tumor progression and aggressiveness. Importantly, we are the first to demonstrate that the LINC00115/miR-489-3p axis is markedly linked to CRC cell proliferation, migration, and invasion, which was achieved by interacting with the PI3K/Akt/mTOR signaling pathway. The findings from our study open up a new understanding of CRC and identify a promising biomarker and therapeutic target for enhancing CRC treatment.

However, limitations of the present study should also be noticed. First, our conclusions were based on the examination on cell lines and may not reflect the exact process in the organism; more *in vivo* researches need to be carried out in further study. Second, further prospective studies with eligibility criteria applicable to clinical trials are needed to confirm our results that LINC00115 exerts as a promising prognostic marker for CRC.

## Data Availability Statement

All data that support the findings of this study are available from the corresponding author upon reasonable request.

## Ethics Statement

The studies involving human participants were reviewed and approved by the Medical Ethics Committee of the Affiliated Cancer Hospital of Zhengzhou University, Henan Cancer Hospital. The patients/participants provided their written informed consent to participate in this study. Written informed consent was obtained from the individual(s) for the publication of any potentially identifiable images or data included in this article.

## Author Contributions

WF and JZ conceived and designed the experiments and wrote the manuscript. WF, BL, DX, and KC performed the experiments. WF, BL, and JW analyzed the data. HZ and YL contributed to the reagents, materials, and tools. All authors read and approved the final manuscript.

## Conflict of Interest

The authors declare that the research was conducted in the absence of any commercial or financial relationships that could be construed as a potential conflict of interest.

## Footnotes

1 http://starbase.sysu.edu.cn/

## References

[B1] Bahrami A. Khazaei M. Hasanzadeh M. ShahidSales S. Joudi Mashhad M. Farazestanian M. (2018). Therapeutic potential of targeting PI3K/AKT pathway in treatment of colorectal cancer: rational and progress. *J. Cell. Biochem.* 119 2460–2469. 10.1002/jcb.25950 28230287

[B2] Cech T. R. Steitz J. A. (2014). The noncoding RNA revolution-trashing old rules to forge new ones. *Cell* 157 77–94. 10.1016/j.cell.2014.03.008 24679528

[B3] Chandra Gupta S. Nandan Tripathi Y. (2017). Potential of long non-coding RNAs in cancer patients: from biomarkers to therapeutic targets. *Int. J. Cancer* 140 1955–1967. 10.1002/ijc.30546 27925173

[B4] Chen W. Sun K. Zheng R. Zeng H. Zhang S. Xia C. (2018). Cancer incidence and mortality in China, 2014. *Chin. J. Cancer Res.* 30 1–12. 10.21147/j.issn.1000-9604.2018.01.01 29545714 PMC5842223

[B5] Chen X. Dong H. Liu S. Yu L. Yan D. Yao X. (2017). Long noncoding RNA MHENCR promotes melanoma progression via regulating miR-425/489-mediated PI3K-Akt pathway. *Am. J. Transl. Res.* 9 90–102. 28123636 PMC5250706

[B6] Chen X. Wang Y. W. Xing A. Y. Xiang S. Shi D. B. Liu L. (2016). Suppression of SPIN1-mediated PI3K-Akt pathway by miR-489 increases chemosensitivity in breast cancer. *J. Pathol.* 239 459–472. 10.1002/path.4743 27171498

[B7] Coppede F. Lopomo A. Spisni R. Migliore L. (2014). Genetic and epigenetic biomarkers for diagnosis, prognosis and treatment of colorectal cancer. *World J. Gastroenterol.* 20 943–956. 10.3748/wjg.v20.i4.943 24574767 PMC3921546

[B8] Dai X. Kaushik A. C. Zhang J. (2019). The emerging role of major regulatory RNAs in cancer control. *Front. Oncol.* 9:920. 10.3389/fonc.2019.00920 31608229 PMC6771296

[B9] Dekker E. Tanis P. J. Vleugels J. L. A. Kasi P. M. Wallace M. B. (2019). Colorectal cancer. *Lancet* 394 1467–1480. 10.1016/S0140-6736(19)32319-0 31631858

[B10] Duan G. Zhang C. Xu C. Xu C. Zhang L. Zhang Y. (2019). Knockdown of MALAT1 inhibits osteosarcoma progression via regulating the miR34a/cyclin D1 axis. *Int. J. Oncol.* 54 17–28. 10.3892/ijo.2018.4600 30365098 PMC6254999

[B11] Esteller M. (2011). Non-coding RNAs in human disease. *Nat. Rev. Genet.* 12 861–874. 10.1038/nrg3074 22094949

[B12] Hong S. N. (2018). Genetic and epigenetic alterations of colorectal cancer. *Intest. Res.* 16 327–337. 10.5217/ir.2018.16.3.327 30090031 PMC6077299

[B13] Hua H. Kong Q. Zhang H. Wang J. Luo T. Jiang Y. (2019). Targeting mTOR for cancer therapy. *J. Hematol. Oncol.* 12:71. 10.1186/s13045-019-0754-1 31277692 PMC6612215

[B14] Huang S. Xu Y. Ge X. Xu B. Peng W. Jiang X. (2019). Long noncoding RNA NEAT1 accelerates the proliferation and fibrosis in diabetic nephropathy through activating Akt/mTOR signaling pathway. *J. Cell. Physiol.* 234 11200–11207. 10.1002/jcp.27770 30515796

[B15] Jiang B. Hailong S. Yuan J. Zhao H. Xia W. Zha Z. (2018). Identification of oncogenic long noncoding RNA SNHG12 and DUXAP8 in human bladder cancer through a comprehensive profiling analysis. *Biomed. Pharmacother.* 108 500–507. 10.1016/j.biopha.2018.09.025 30243082

[B16] Kapranov P. Willingham A. T. Gingeras T. R. (2007). Genome-wide transcription and the implications for genomic organization. *Nat. Rev. Genet.* 8 413–423. 10.1038/nrg2083 17486121

[B17] Li Y. Ma X. Wang Y. Li G. (2017). miR-489 inhibits proliferation, cell cycle progression and induces apoptosis of glioma cells via targeting SPIN1-mediated PI3K/AKT pathway. *Biomed. Pharmacother.* 93 435–443. 10.1016/j.biopha.2017.06.058 28666210

[B18] Liu L. Wang H. J. Meng T. Lei C. Yang X. H. Wang Q. S. (2019). lncRNA GAS5 inhibits cell migration and invasion and promotes autophagy by targeting miR-222-3p via the GAS5/PTEN-signaling pathway in CRC. *Mol. Ther. Nucleic Acids* 17 644–656. 10.1016/j.omtn.2019.06.009 31400607 PMC6698928

[B19] Liu Q. Yang G. Qian Y. (2017). Loss of MicroRNA-489-3p promotes osteosarcoma metastasis by activating PAX3-MET pathway. *Mol. Carcinog.* 56 1312–1321. 10.1002/mc.22593 27859625

[B20] Liu X. Cui L. Hua D. (2018). Long noncoding RNA XIST regulates miR-137-EZH2 axis to promote tumor metastasis in colorectal cancer. *Oncol. Res.* 27 99–106. 10.3727/096504018X15195193936573 29495975 PMC7848292

[B21] Liu Y. Zhou J. Wang S. Song Y. Zhou J. Ren F. (2019). Long non-coding RNA SNHG12 promotes proliferation and invasion of colorectal cancer cells by acting as a molecular sponge of microRNA-16. *Exp. Ther. Med.* 18 1212–1220. 10.3892/etm.2019.7650 31316616 PMC6601377

[B22] Lu S. R. Li Q. Lu J. L. Liu C. Xu X. Li J. Z. (2018). Long non-coding RNA LINC01503 promotes colorectal cancer cell proliferation and invasion by regulating miR-4492/FOXK1 signaling. *Exp. Ther. Med.* 16 4879–4885. 10.3892/etm.2018.6775 30542444 PMC6257603

[B23] Meng L. H. Zheng X. F. (2015). Toward rapamycin analog (rapalog)-based precision cancer therapy. *Acta Pharmacol. Sin.* 36 1163–1169. 10.1038/aps.2015.68 26299952 PMC4648176

[B24] Okugawa Y. Grady W. M. Goel A. (2015). Epigenetic alterations in colorectal cancer: emerging biomarkers. *Gastroenterology* 149 1204.e12–1225.e12. 10.1053/j.gastro.2015.07.011 26216839 PMC4589488

[B25] Siegel R. L. Miller K. D. Fedewa S. A. Ahnen D. J. Meester R. G. S. Barzi A. (2017). Colorectal cancer statistics, 2017. *CA Cancer J. Clin.* 67 177–193. 10.3322/caac.21395 28248415

[B26] Siegel R. L. Miller K. D. Jemal A. (2019). Cancer statistics, 2019. *CA Cancer J. Clin.* 69 7–34. 10.3322/caac.21551 30620402

[B27] Sun F. Liang W. Tang K. Hong M. Qian J. (2019). Profiling the lncRNA-miRNA-mRNA ceRNA network to reveal potential crosstalk between inflammatory bowel disease and colorectal cancer. *PeerJ* 7:e7451. 10.7717/peerj.7451 31523496 PMC6714963

[B28] Tang J. Yu B. Li Y. Zhang W. Alvarez A. A. Hu B. (2019). TGF-beta-activated lncRNA LINC00115 is a critical regulator of glioma stem-like cell tumorigenicity. *EMBO Rep.* 20:e48170. 10.15252/embr.201948170 31599491 PMC6893290

[B29] Tsang C. K. Qi H. Liu L. F. Zheng X. F. (2007). Targeting mammalian target of rapamycin (mTOR) for health and diseases. *Drug Discov. Today* 12 112–124. 10.1016/j.drudis.2006.12.008 17275731

[B30] Wang M. Hu H. Wang Y. Huang Q. Huang R. Chen Y. (2019). Long non-coding RNA TUG1 mediates 5-fluorouracil resistance by acting as a ceRNA of miR-197-3p in colorectal cancer. *J. Cancer* 10 4603–4613. 10.7150/jca.32065 31528224 PMC6746119

[B31] Wang X. Lan Z. He J. Lai Q. Yao X. Li Q. (2019). LncRNA SNHG6 promotes chemoresistance through ULK1-induced autophagy by sponging miR-26a-5p in colorectal cancer cells. *Cancer Cell. Int.* 19:234. 10.1186/s12935-019-0951-6 31516391 PMC6734319

[B32] Wang Y. Jiang F. Xiong Y. Cheng X. Qiu Z. Song R. (2019). LncRNA TTN-AS1 sponges miR-376a-3p to promote colorectal cancer progression via upregulating KLF15. *Life Sci.* 244:116936. 10.1016/j.lfs.2019.116936 31610194

[B33] Yu G. J. Sun Y. Zhang D. W. Zhang P. (2019). Long non-coding RNA HOTAIR functions as a competitive endogenous RNA to regulate PRAF2 expression by sponging miR-326 in cutaneous squamous cell carcinoma. *Cancer Cell. Int.* 19:270. 10.1186/s12935-019-0992-x 31649487 PMC6805682

[B34] Zhang L. Wang Q. Wang F. Zhang X. Zhang L. Tang Y. (2018). LncRNA LINC01446 promotes glioblastoma progression by modulating miR-489-3p/TPT1 axis. *Biochem. Biophys. Res. Commun.* 503 1484–1490. 10.1016/j.bbrc.2018.07.067 30029885

[B35] Zhu Y. Zhang X. Qi L. Cai Y. Yang P. Xuan G. (2016). HULC long noncoding RNA silencing suppresses angiogenesis by regulating ESM-1 via the PI3K/Akt/mTOR signaling pathway in human gliomas. *Oncotarget* 7 14429–14440. 10.18632/oncotarget.7418 26894862 PMC4924726

